# CD44 drives aggressiveness and chemoresistance of a metastatic human osteosarcoma xenograft model

**DOI:** 10.18632/oncotarget.23125

**Published:** 2017-12-09

**Authors:** Lisa Mayr, Christine Pirker, Daniela Lötsch, Sushilla Van Schoonhoven, Reinhard Windhager, Bernhard Englinger, Walter Berger, Bernd Kubista

**Affiliations:** ^1^ Institute of Cancer Research and Comprehensive Cancer Center, Department of Medicine I, Medical University Vienna, 1090 Vienna, Austria; ^2^ Department of Orthopaedics, Medical University Vienna, 1090 Vienna, Austria

**Keywords:** CD44, osteosarcoma, metastasis, metastatic model, cilengitide

## Abstract

**Background:**

Osteosarcoma is the most common primary malignant bone tumor with a 5 year survival rate of up to 70%. However, patients with metastatic disease have still a very poor prognosis. Osteosarcoma metastasis models are essential to develop novel treatment strategies for advanced disease.

**Methods:**

Based on a serial transplantation approach, we have established a U-2 OS osteosarcoma xenograft model with increased metastatic potential and compared it to other metastatic osteosarcoma models from international sources. Subclones with differing invasive potential were compared for genomic gains and losses as well as gene expression changes by several bioinformatic approaches. Based on the acquired results, the effects of a shRNA-mediated *CD44* mRNA knockdown on migration, invasion and chemosensitivity were evaluated.

**Results:**

The *CD44* gene was part of an amplified region at chromosome 11p found in both U-2 OS subclones with enhanced metastatic potential but not in parental U-2 OS cells, corresponding with distinct CD44 overexpression. Accordingly, shRNA-mediated *CD44* knockdown significantly attenuated osteosarcoma cell migration, invasion, and viability especially in the metastatic subclones of U-2 OS and Saos-2 cells. Metastatic subclones generally were hypersensitive against the integrin inhibitor cilengitide paralleled by alterations in integrin expression pattern following *CD44* knock-down. Additionally, attenuation of CD44 expression sensitized these cell models against osteosarcoma chemotherapy with doxorubicin but not methotrexate and cisplatin.

**Conclusions:**

The osteosarcoma xenograft models with increased metastatic potential developed in this study can be useful for identification of mechanisms driving metastasis and resistance towards clinically used and novel therapeutic regimens.

## INTRODUCTION

Osteosarcoma (OS) is the most common primary malignant bone tumor. The introduction of multimodal chemotherapy together with surgical resection improved cure rates from less than 30% up to 70% in the past decades. However, about 30% of cases develop pulmonary metastases, and for these patients treatment options are very limited resulting in poor overall survival [1, 2]. Several possible prognostic factors for metastatic spread and survival in OS have been evaluated in the past, however most of them failed to prove their value in the clinical setting [1, 3–5]. One of the most important prognostic factors is the development of lung metastases. So far, mechanisms that lead to metastatic spread and determine the aggressiveness of OS cells remain unclear. Characterizing OS cells according to their metastatic potential would allow stratification of patients who may benefit from a more effective and customized therapy. Due to the very low incidence of OS, the development of new therapeutic strategies is very challenging and requires time consuming multi-center studies [6]. This is the reason why in the last decades only very few new agents could be evaluated and no further progress in OS treatment has been achieved [7, 8].

It is therefore of utmost importance to develop metastatic OS cell models that allow identification of therapeutic targets and pre-testing of respective treatment approaches especially for this patient subgroup. In this study we have developed a xenograft model of human OS and evaluated genetic alterations that occurred in OS cells metastasizing to the lungs. Among others we identified a significant increase in the expression of *CD44* in the metastatic OS cells. CD44 is a surface glycoprotein encoded by the *CD44* gene on chromosome 11p13. *CD44* mRNA can undergo alternative splicing resulting in *CD44* standard (CD44s) and *CD44* variant (CD44v) isoforms. CD44s is expressed on most mesenchymal and hematopoietic cells and plays a major regulatory role in the interaction between cells and the extracellular matrix (ECM). Several previous studies demonstrated altered *CD44* expression in various different malignancies [9–12]. Furthermore, *CD44* expression has been linked to the metastatic potential and was evaluated as a possible prognostic marker in OS [10, 13–16].

Aim of this study was to develop a model for metastasizing human OS and to detect molecular alterations differing between the original cell line and the respective subclones with higher metastatic potential. CD44 was confirmed as a major player of OS invasive and metastatic potential and, additionally, as a chemoresistance mechanism against the standard OS drug doxorubicin.

## RESULTS

### Establishment of an aggressive and higher metastatic OS cell model

**Figure 1:**
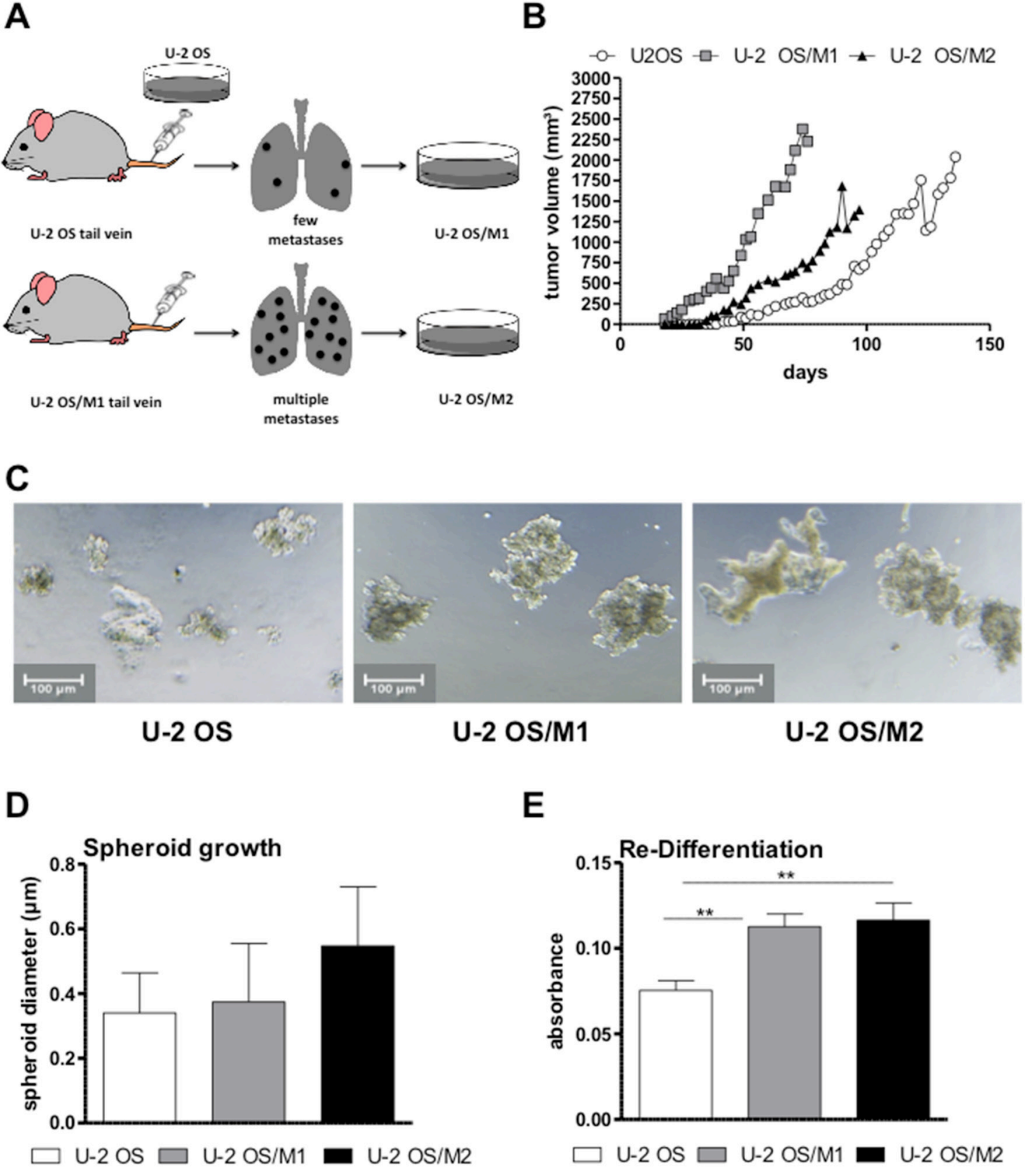
Generation of a hyper-metastatic OS cell model **(A)** The serial transplantation strategy used to establish hyper-metastatic OS cell models is depicted (compare Material and Methods). **(B)** From each OS cell model, 1x10^6^ cells were subcutaneously xenografted and tumor growth was measured every second day by caliper. **(C)** Spheroid formation was tested by seeding OS cells in ultra-low attachment plates in spheroid growth medium. Representative photomicrographs were taken after 96 hours. **(D)** Spheroid size was analysed with Image-J software. **(E)** The re-differentiation potential was tested by re-plating sphere-derived single cells in 24-well plates with IMDM medium. After 7 days cells were fixed, stained and further analysed. Two experiments performed in duplicates are shown. One-way ANOVA with Bonferroni’s post hoc test; ^**^ p < 0.01.

By performing a serial transplantation approach based on re-establishment of cell lines from OS lung metastases after tail vein injection (strategy outlined in Figure 1A), we have successfully established a hyper-metastatic OS model. The primary OS cell line U-2 OS induced a significantly lower number of lung metastases in contrast to U-2 OS/M1 and U-2 OS/M2, which caused multiple metastases. When grown as subcutaneous xenografts, all three cell lines were tumorigenic. Distinct differences in local tumor aggressiveness could be observed. The two metastatic cell models were characterized by a more rapid tumor growth and a greater tumor volume when compared to U-2 OS (Figure 1B). However, despite their massive lung colonisation after tail vein injection, U-2 OS/M1 and U-2 OS/M2 failed to form metastases from subcutaneous xenografts (data not shown). To test stemness properties, the cell lines were grown under non-adherent and serum-free conditions. The two metastatic subclones formed more and slightly larger spheroids when compared to U-2 OS cells (Figure 1C and 1D). Furthermore, the metastatic subclones were able to re-differentiate at a higher potency than the parental cells (Figure 1E).

### Gene dose and expression changes associated with enhanced metastatic potential

**Figure 2:**
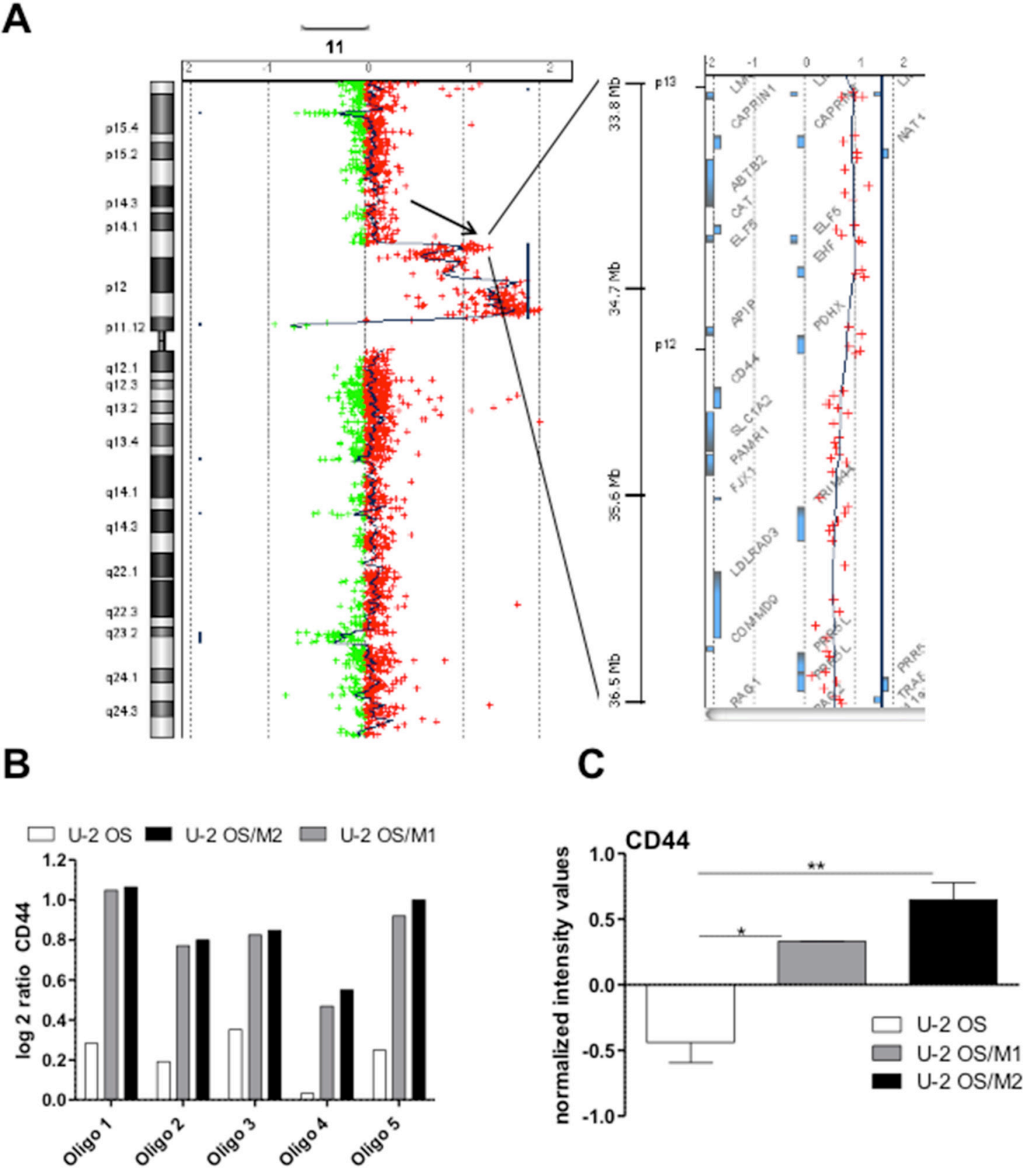
Altered gene dose and gene expression levels of *CD44* in hyper-metastatic OS subclones **(A)** Gains/losses within chromosome 11 in hyper-metastatic U-2 OS/M1 as compared to parental U-2 OS cells were detected by indirect aCGH. The left panel displays entire chromosome 11 with a major gain (peak to the right side) of the region 11p11.12 to 11p14.1 including the *CD44* gene locus (arrow). The right panel zooms into the *CD44* gene locus on 11p13 with the genes located in the respective region depicted as bars. **(B)** Log2 gene dose ratios of U-2 OS-derived DNA as compared to non-malignant, diploid reference DNA as well as of U-2 OS/M1 and U-2 OS/M2 as compared to the parental U-2 OS DNA are depicted for the five *CD44* oligonucleotides on the microarray. **(C)** Whole genome gene expression analysis: normalized intensity values for *CD44* of the three indicated cell models are shown. The data are evaluated with the GeneSpring software. One-way ANOVA with Bonferroni’s post hoc test; ^*^ p < 0.05; ^**^ p < 0.01.

**Figure 3:**
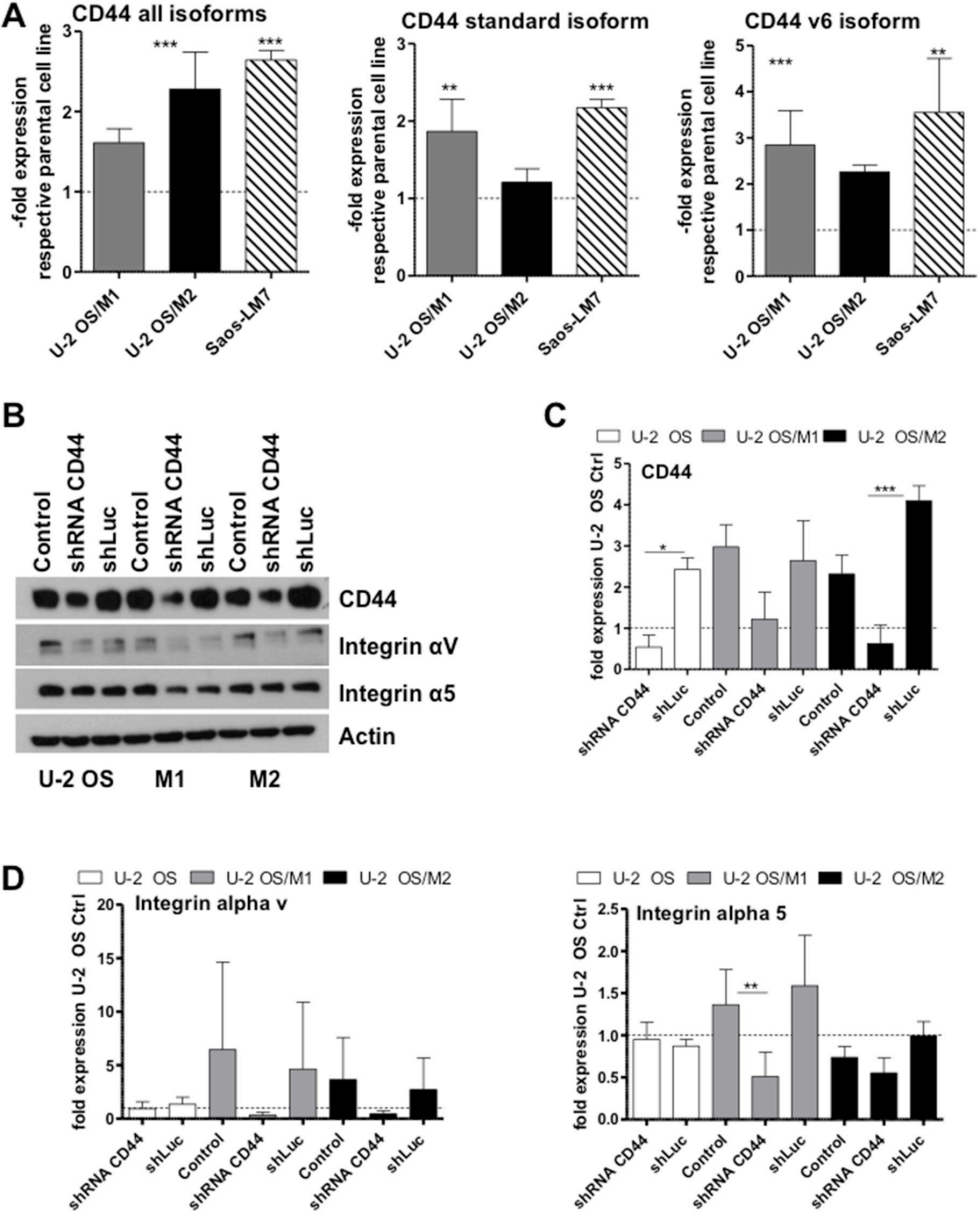
*CD44* mRNA and protein expression levels in OS cells and impact of *CD44* knock-down on cell adhesion molecules **(A)** *CD44* mRNA expression was determined by real-time PCR with the indicated primer sets. Two experiments were performed in triplicates. **(B-D)** Protein expression levels were determined by Western blot analysis in the indicated cell lines either untransfected (control) or transfected with a shRNA targeting *CD44* mRNA (shRNA *CD44*) or a shLuc vector control. One out of three experiments is shown representatively. (C, D) Numbers depict densitometric quantification of the Western blots (ImageJ Software) for the indicted genes and data are in all cases given as expression levels relative to the untransfected control of each OS cell line. Values are means of three experiments. One-way ANOVA with Bonferroni’s post hoc test; ^*^ p < 0.05; ^**^ p < 0.01; ^***^ p < 0.001.

For determination of genome wide gene copy number alterations aCGH was performed. All models showed changes already described for human OS including gains at chromosomes 8q and 17q, as well as losses at chromosomes 6q, 13q, and 17p. [17] (Supplementary Figure 1 for the parental cell line). When comparing the hyper-metastatic sublines to the parental line by indirect aCGH, a prominent gain at chromosome 11p13 harboring the *CD44* gene was observed (Figure 2A, Supplementary Figures 2, 3, and 4). In contrast to the parental line, where only a low level gain was seen, both hyper-metastatic models showed distinct amplification of all 5 *CD44* oligonucleotides (Figure 2B). In addition, several changes including genes associated with migration and metastasis were detected, e.g. gain of *AKT3* (1q43-44), *RAB31* (18p11.22), *SRC* (20q11.23) and loss of *RAP1A* (1p13.2), *TNS3* (7p12.3), and *RAB27A* (15q21.3) (Supplementary Figures 2 and 3). No differences between U-2 OS/M1 and U-2 OS/M2 at the genomic level could be observed (Supplementary Figure 5). In a further approach we investigated gene expression alterations at the mRNA level by performing whole genome gene expression arrays with all three OS models. At a comparably low stringency (p<0.001; fold change >1.5) our analysis proved distinct upregulation of *CD44* mRNA levels in the metastatic OS cell models as compared to U-2 OS cells (Figure 2C). In order to validate *CD44* mRNA overexpression, we performed real-time PCR experiments. While the “*CD44* all” primer pair detects all splice variants of the gene, the “*CD44* standard” primers can only amplify the *CD44* standard variant lacking the entire variable region and the “*CD44*v6” primers amplify only the splice variant *CD44v6* (Figure 3A). *CD44* mRNA expression was significantly higher in the hyper-metastatic subclones when compared to the respective parental cell line (U-2 OS). To confirm our findings we analysed *CD44* mRNA expression in an additional hyper-metastatic OS cell model of international origin (Saos-LM7) [5]. Saos-LM7 showed a significantly increased *CD44* mRNA expression when compared to the parental cell line Saos-2 (Figure 3A). All metastatic OS cell models had a higher expression level of the *CD44* standard variant when compared to the parental cell model. The *CD44v6* variant was significantly upregulated in all three metastatic OS cell models, again with the highest expression levels detected in Saos-LM7.

### Impact of *CD44* knock-down on expression of cell adhesion molecules

To test a causative role of *CD44* overexpression in the invasive phenotype, we performed a shRNA-mediated gene knockdown of *CD44* (Figure 3B and 3C). U-2 OS/M1 and U-2 OS/M2 exhibited distinctly higher CD44 protein expression levels when compared to U-2 OS cells which could be significantly down-regulated by the shRNA approach. Furthermore, CD44 protein expression was only detectable in the hyper-metastatic cell model Saos-LM7 in contrast to the respective parental cell line Saos-2 (Supplementary Figure 6). Of note, *CD44* gene knockdown distinctly decreased integrin αν and integrin α5 protein expression levels especially in the hyper-metastatic sublines (Figure 3D). Gene Set Enrichment Analysis (GSEA) indicated an enrichment of the Biocarta term “integrin pathway” in the hyper-metastatic cell lines U-2 OS/M1 and U-2 OS/M2 when compared to the parental cell line (data shown in Supplementary Figure 7A). Integrin αν and α5 mRNA expression levels were significantly decreased in the cells with shRNA-mediated *CD44* gene knockdown when compared to shLuc vector control in our OS cell models (Supplementary Figure 7B). Furthermore, treatment with the RGD-peptide integrin inhibitor cilengitide [18] decreased viability of all hyper-metastatic cell models (U-2 OS/M1, U-2 OS/M2 and Saos-LM7) at significantly higher potency as compared to the respective parental cell lines (Supplementary Figure 7C).

### CD44 supports OS cell migration and invasion

**Figure 4:**
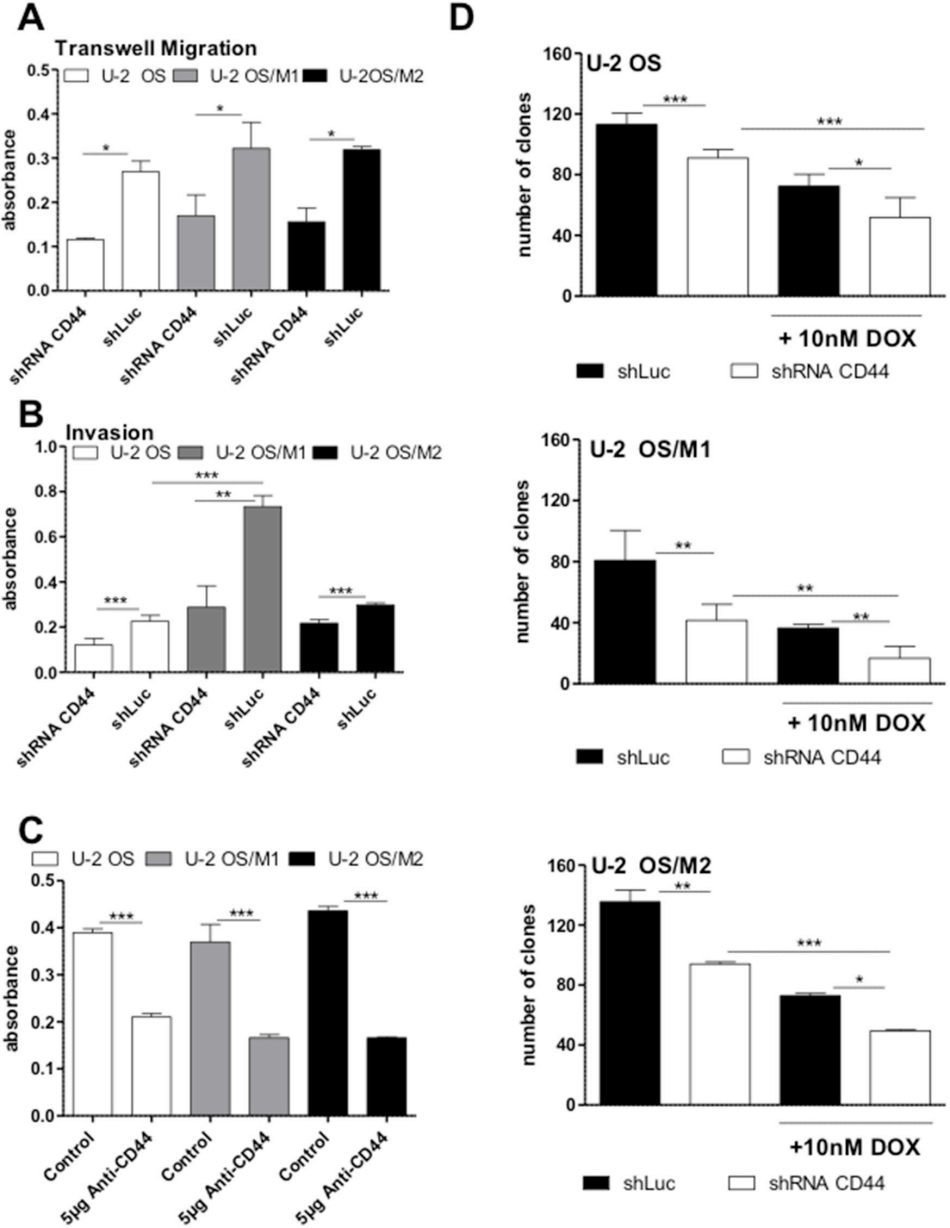
Impact of *CD44* gene knockdown on OS cell migration, invasion, clonogenicity and chemotherapy resistance **(A)** Transwell migration assays were performed for 48 hours under standard culture conditions. Cell migration to the lower side of the membrane was determined by densitometric quantification of crystal violet staining. Three experiments were performed in duplicates. **(B)** Invasion assays were performed with matrigel covered filters for 48 hours and evaluated as under (A). Three experiments in duplicates were performed. **(C)** Clonogenic assays were performed under standard culture conditions with and without 5 μg anti-CD44 antibody for 7 days. Densitometric quantification of the crystal violet stained cell clones are shown. Two experiments were performed in duplicates. **(D)** Clonogenic assays were performed under standard culture conditions with and without 10 nM doxorubicin for 7 days. Means of three experiments in duplicates are depicted. One-way ANOVA with Bonferroni’s post hoc test; ^*^ p < 0.05; ^**^ p < 0.01; ^***^ p < 0.001.

Consequently, we tested the impact of *CD44* knock-down on cell migration and invasion. The migratory potential was slightly higher in our hyper-metastatic cell lines when compared to the primary OS cell line U-2 OS. Furthermore, U-2 OS/M1 as well as to a lower extent U-2 OS/M2 exerted a distinctly higher invasive behavior. *CD44* gene knockdown led to a significantly decreased migratory potential especially of the hyper-metastatic OS cell models (Figure 4A and Supplementary Figure 8A). Moreover, the *CD44* gene knockdown led to a significantly decreased invasiveness in all U-2 OS cell lines, especially in the highly aggressive U-2 OS/M1 subclone (Figure 4B). In the metastatic Saos-LM7 and K7M2 sublines - but not the parental Saos-2 cells - invasiveness was moderately but significantly decreased following *CD44* knockdown (Supplementary Figure 8B).

### CD44 supports clonogenicity and chemotherapy resistance in OS cells

The short-term exposure experiment (MTT assay) revealed markedly decreased IC_50_ values for doxorubicin in all OS cell models following *CD44* gene knockdown when compared to the scrambled vector control (Table 1). Furthermore, in the long-term exposure experiment (clonogenic assay) blocking of CD44 with either 5 μg of an anti-CD44 antibody (Figure 4C and Supplementary Figure 8C) or by shRNA (Figure 4D) significantly decreased clonogenicity in all three OS cell models as compared to the respective controls. Combined application of *CD44* gene knockdown and 10nM doxorubicin showed a weak synergistic effect in all three OS cell models (Figure 4D). In contrast, *CD44* knockdown did not sensitize any OS cell model against methotrexate and cisplatin (Supplementary Figure 9).

**Table 1:** Anticancer activity of doxorubicin against OS cell models and impact of a CD44 knockdown

	Doxorubicin (IC_50_; nM)		Fold sensitization
Cell line	shLuc	shRNA CD44	ShLuc/ shRNA CD44
U-2 OS	52.3 ± 0.2	39.4 ± 0.1	1.33
U-2 OS/M1	39.2 ± 0.1	13.9 ± 0.1	2.82
U-2 OS/M2	87.6 ± 0.1	56.3 ± 0.05	1.56

### CD44 is related to an increased *in vivo* aggressiveness

**Figure 5:**
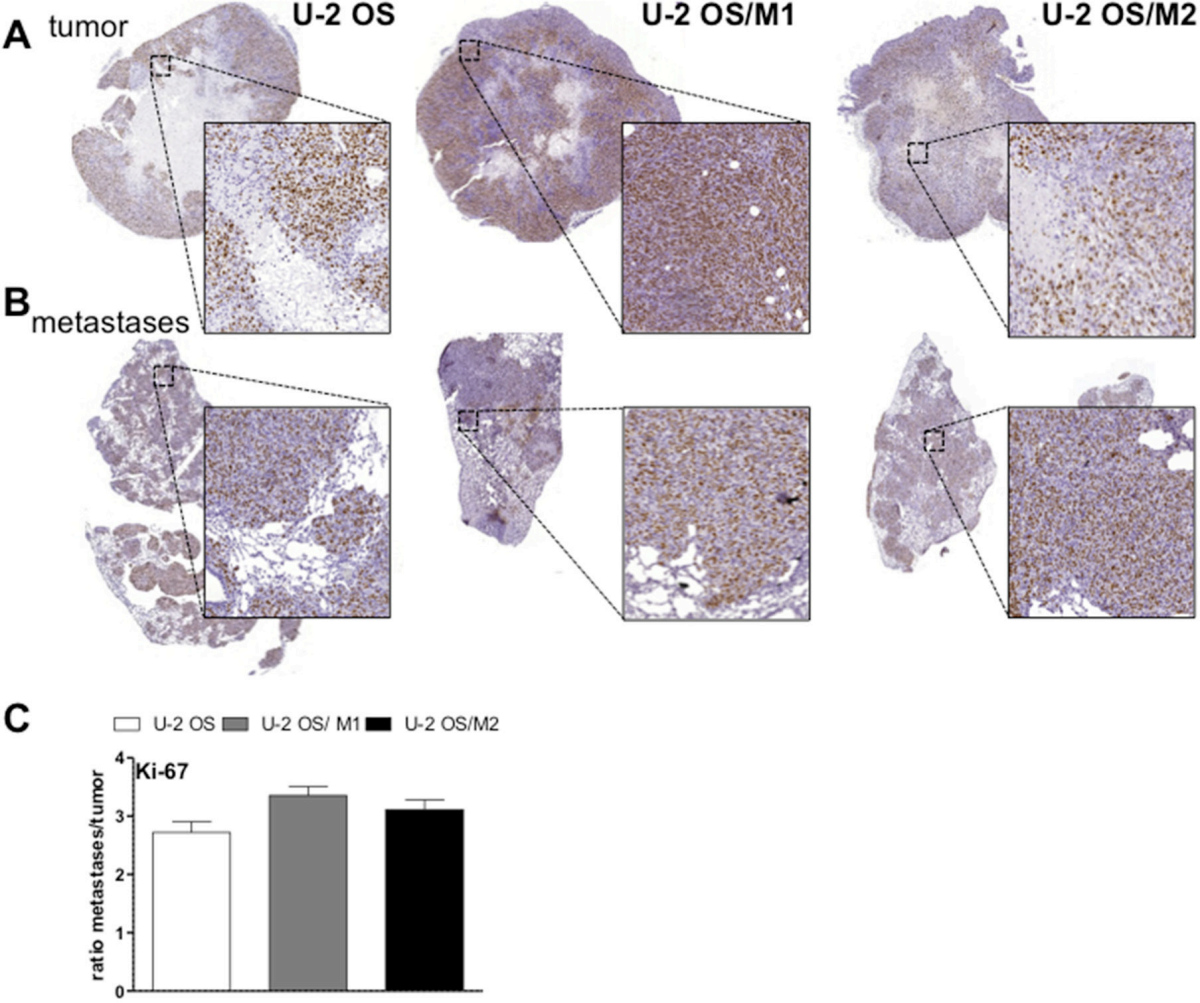
Ki-67 expression in lung metastases and subcutaneous tumors of xenografted OS cells Tissue sections of subcutaneous OS xenografts **(A)** and tail vein injection-induced lung metastases **(B)** were analysed immunohistochemically. Sections were stained for the proliferation marker Ki-67. **(C)** Quantification of the proportion of tumor cells positively stained for Ki-67 was analyzed by Definiens software. Values are given as percentage positively-stained tumor cells in metastases divided by subcutaneous tumors (ratio).

**Figure 6:**
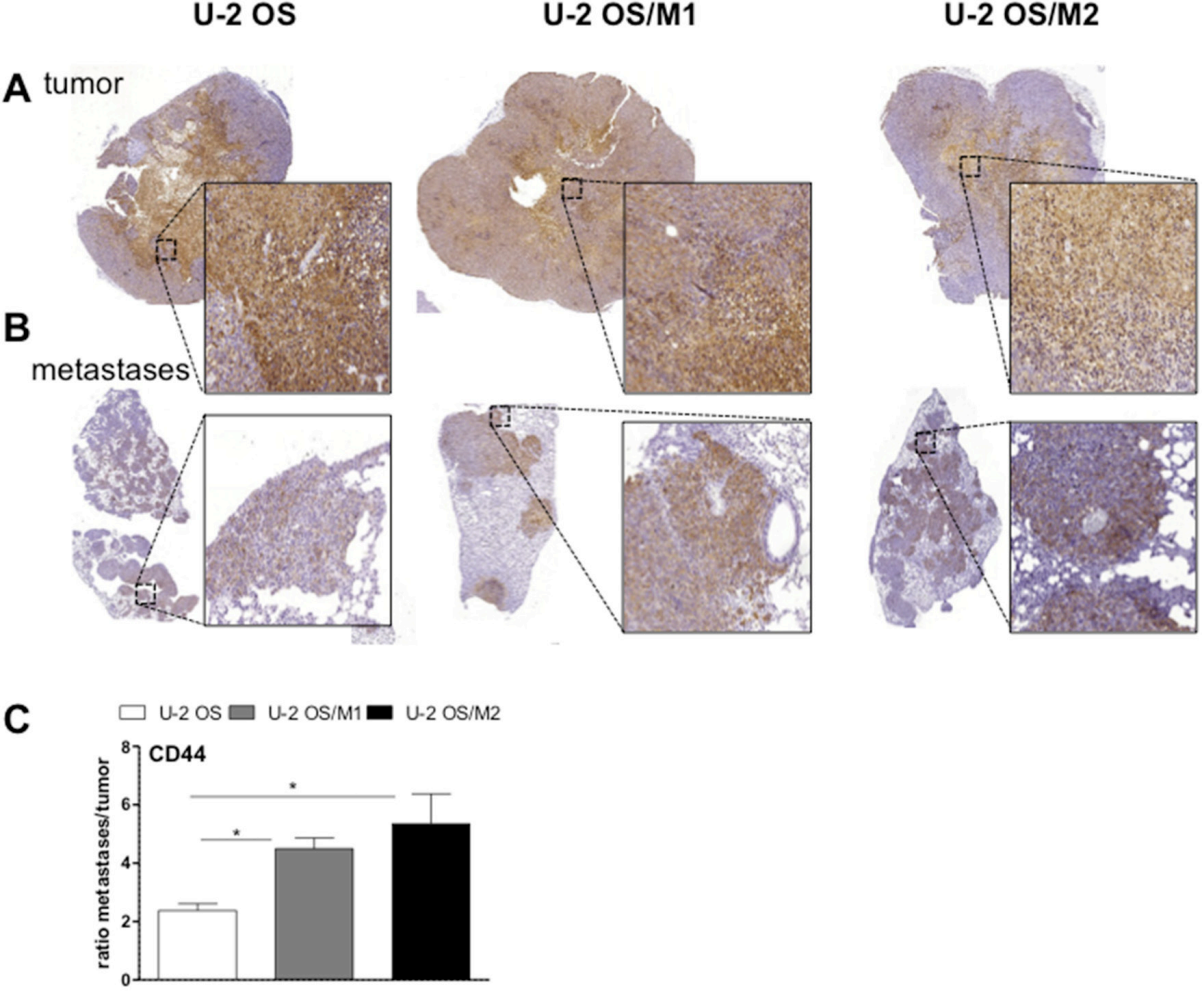
CD44 expression in lung metastases and subcutaneous tumors of xenografted OS cells Tissue sections of subcutaneous OS xenografts **(A)** and tail vein injection-induced lung metastases **(B)** were analysed immunohistochemically. Sections were stained for CD44. **(C)** Quantification of the proportion of positively stained areas for CD44 analyzed by Definiens software. Values are given as percentage positively-stained areas for CD44 in comparison to the total tissue in metastases divided by subcutaneous tumors (ratio). Students t-test; ^*^ *p* < 0.05.

To test the *in vivo* relevance of *CD44* overexpression in the metastatic process, we performed immunohistochemical stainings of CD44 in subcutaneous tumor xenografts as compared to lung metastases induced by OS cell tail vein injection. Expression of the proliferation marker Ki-67 was significantly enriched (around 3-fold enhanced percentage of positively stained cells) in metastases as compared to primary tumors without significant differences between the three U-2 OS cell models (Figure 5). Metastases were generally enriched in CD44 staining as compared to the subcutaneous xenografts (Figure 6A and 6B), but this effect was distinctly stronger in the U-2 OS/M1 and U-2 OS/M2 subclones (Figure 6C). Saos-LM7 led to a significantly enhanced number of metastatic lesions as compared to parental Saos-2 cells following tail vein injection. Additionally, CD44 expression was markedly enriched in the metastases of Saos-2 and the hyper-metastatic subclone Saos-LM7 when compared to the respective subcutaneous tumors (Supplementary Figure 10).

## DISCUSSION

During the last decades, survival of OS did not substantially improve. Especially for patients with local relapse or metastatic disease the prognosis is still very poor and treatment options are limited [7]. OS-related death is due to metastatic spread to the lungs rather than primary tumor or local relapse and therefore development of lung metastasis is one of the most important prognostic factors [1]. Thus, dissection of mechanisms driving systemic spread and the development of novel therapeutic strategies that target metastasizing OS is of utmost importance. So far, therapeutic options specifically targeting recurrent and metastatic disease as well as biomarkers to identify patients who could benefit from a more aggressive and customized therapy are missing. Reliable *in vitro* and *in vivo* models for metastatic OS are urgently needed to allow conclusive evaluation of innovative therapy targets, experimental compounds and combination strategies.

Despite the need for a more effective treatment to prevent the dissemination of OS, still little is known about the mechanisms that drive development of lung metastasis. Hence, we aimed in this study to establish a relevant, preclinical xenograft mouse model of highly metastatic human OS by serial *in vivo* passages. We successfully developed and characterized two hyper-metastatic sublines derived from lung metastases of parental U-2 OS cells following tail vein injection. Furthermore, we conducted global gene dose and gene expression analyses to identify possible factors that promote the development of lung metastasis. We found several genetic alterations that have been linked to tumor aggressiveness and metastasis in the past. Among other factors, we identified *CD44* gene dose gains and overexpression of different *CD44* mRNA variants in the highly metastatic cell lines U-2 OS/M1, U-2 OS/M2 and Saos-LM7 compared to the parental cell line U-2 OS and Saos-2 with lower metastatic potential, respectively. This is in line with previous studies that demonstrated an increase of *CD44* expression in cells derived from metastatic lesions compared to primary tumor cells [10].

CD44, a transmembrane surface protein, is a receptor for hyaluronic acid and regulates the interaction of cells with the extracellular matrix (ECM) [19]. A large variety of isoforms exist, generated by alternative splicing of up to 10 exons (*CD44v1-CD44v10*). Some of these isoforms have been linked to tumor invasion, metastasis, and prognosis [20]. Accordingly, the isoform *CD44v6* was distinctly overexpressed especially in the cell model U-2 OS/M1 and Saos-LM7 with high invasive potential and aggressiveness *in vivo*. Furthermore, CD44 is an important cancer stem cell (CSC) surface marker. CSC have the ability to self-renew and differentiate into various cell types. Many studies support the important role of CSC in tumor initiation and metastasis as well as therapy resistance [21]. In a previous study increased CD44 expression has been linked to sphere formation and metastatic potential in OS [10]. Accordingly, we found an increased spheroid formation rate and re-differentiation capability of the metastatic subclones compared to the parental U-2 OS cells *in vitro* as well as enhanced tumorigenicity *in vivo*, all parameters reflecting stemness potential.

The relevance of CD44 and its isoforms as a prognostic factor has been studied for patients with several solid tumors including breast, colon, lung, and oesophageal cancer, however its role in OS remains controversial [13]. Based on enhanced gene copy number as well as gene expression detected by aCGH and gene expression analyses, respectively, we decided to evaluate the role of *CD44* overexpression in tumor cell behavior and resistance to standard chemotherapy *in vitro*. We found an increased migratory potential and enhanced tumor invasion of metastatic OS cells expressing high levels of CD44. The crucial role of CD44 in this invasive phenotype was confirmed by a shRNA-mediated gene knock-down which significantly decreased the migratory and invasive potential especially in the hyper-metastatic subclones (U-2 OS/M1, U-2 OS/M2, Saos-LM7 and K7M2). These findings are in accordance with previous studies that demonstrated the central role of CD44 expression in adhesion, migration and invasion of OS cells [22]. The exact mechanisms by which CD44 enhances cell motility and metastatic potential are complex and multifaceted. Recently, it was demonstrated that interaction with several components of the microenvironment - including besides hyaluronic acid also many other ECM components like collagens, osteopontin, cytokines and growth factors [21] - are essential for the role of CD44 in driving metastatic dissemination [23]. Interestingly, in our hyper-metastatic cell models knock-down of *CD44* also had a distinct impact on the levels of the oncogenic integrin subunits αν and α5. Moreover, CD44-overexpressing subclones displayed hypersensitivity against integrin blockade by the RGD peptide compound cilengitide [24, 25]. Accordingly, it was recently reported that CD44 supported adhesion of basal-like breast cancer cell to endothelium and fibronectin in an integrin α5β1-dependent manner [26]. This suggests a central role of CD44 in adhesion of OS cells to extracellular matrix components and probably also integrin-mediated survival signals.

In previous reports, CD44 expression has been linked to chemoresistance based on ABC transporter overexpression including ABCB1 and ABCC2 in various tumors especially in the cancer stem cell compartment [21, 27]. We therefore evaluated the effect of doxorubicin, a standard OS chemotherapeutic and substrate for the above-mentioned efflux pumps, on cell viability and clonogenic potential of our hyper-metastatic U-2 OS subclones. Indeed, *CD44* knockdown increased sensitivity to doxorubicin especially in the CD44-overexpressing subclones. This is in agreement with observations by other authors concerning OS cells [28, 29]. In case of T-cell acute lymphoblastic leukaemia, CD44 promoted chemoresistance by supporting enhanced drug efflux [30]. Furthermore, CD44 increased chemoresistance in non-small cell lung cancer by upregulation of ABCC2 [31]. Methotrexate and cisplatin were evaluated to further analyse the CD44-mediated chemoresistance in our OS model. *CD44* gene knockdown in combination with either methotrexate or cisplatin had no sensitizing effect on the tested OS cells. As neither methotrexate nor cisplatin is an ABCB1 substrate, the assumption that CD44 chemoresistance is mediated via ABC transporter overexpression seems likely. Whether the molecular mechanisms underlying the enhanced resistance to doxorubicin in our hyper-metastatic cell subclones is indeed induced by ABC transporter overexpression or rather mediated by integrin-induced survival signals [32] is matter of ongoing investigations.

Summarizing, we have established a human OS xenograft model with high metastatic potential by serial tail vein transplantation and cell line re-establishment. Based on enhanced gene copy number and gene expression, we identified *CD44* upregulation as a major player in tumor cell aggressiveness and resistance to chemotherapy. This new model for metastatic OS could be useful for evaluating advanced therapeutic regimens and customized therapies targeting CD44 in the future.

## MATERIALS AND METHODS

### Cell cultures

The U-2 OS, Saos-2 and K7M2 cell line were obtained from the American Type Culture Collection (ATCC, Manassas, VA). The hyper-metastatic cell model Saos-LM7 was kindly provided by Prof. Eugenie Kleinerman, MD. The metastatic OS cell models U-2 OS/M1 and U-2 OS/M2 were established in our lab by serial tail vein transplantation and cell line re-establishment from lung metastases. U-2 OS cells (1 × 10^5^ cells per mouse), a commonly used OS cell line, were injected via tail vein into 4 SCID/BALBc mice (Harlan Winkelman, Borchen, Germany). Only a few macroscopic lung metastases could be detected. One metastasis was further cultivated and designated U-2 OS/M1. After tail vein injection of the U-2 OS/M1 cell line all mice developed multiple metastases in the lung. One metastasis was cultivated and designated U-2 OS/M2. Also U-2 OS/M2 caused multiple metastases in all mice following tail vein injection. U-2 OS and the metastatic sublines were cultured in IMDM growth medium, Saos-2 in McCoy's 5A, Saos-LM7 in MMP and K7M2 in D10 growth medium supplemented with 10% fetal calf serum (FCS) at 37°C in a 5 % CO_2_ incubator. The U-2 OS cell line was authenticated by array comparative genomic hybridization (44 k human whole genome DNA microarrays; Agilent Technologies, Santa Clara, CA) as published [33] and/or short tandem repeat (STR) fingerprinting before the start of this study.

### Cell viability and cytotoxicity assays

Cells were plated (2 × 10^4^ cells/mL) in 100 μL per well in 96-well plates and allowed to attach for 24 hours. Doxorubicin (0 to 250 nM) or cilengitide (0 to 25μM) were added in 100 μL growth medium with 10% FCS and cells were exposed for 72 hours. The proportion of viable cells was determined by 3-(4,5-dimethylthiazol-2-yl)-2,5-diphenyltetrazolium assay (MTT) following the manufacturer’s recommendations (EZ4U, Biomedica, Vienna, Austria) as published [34]. Cytotoxicity was expressed as IC_50_-values calculated from full dose–response curves (drug concentrations inducing a 50% reduction of the cell number in comparison to the untreated control cells).

### Colony formation assay

500 cells per well were seeded in 500 μL in 24-well plates. Following 24 hours of recovery, 10nM doxorubicin, 0.1μM methotrexate or 0.5μM cisplatin or 5 μg anti-CD44 antibody (ab24504, Abcam, Cambridge, UK) were applied. At day 7 of exposure cells were washed twice with 1xPBS, fixed with methanol at 4°C and stained with crystal violet (10 μg crystal violet per 10 mL PBS). Colonies were counted and evaluated with ImageJ and GraphPad Prism software, respectively, or the crystal violet was dissolved in 2 % SDS and measured with a spectrophotometer at 580 nm absorbance.

### Transwell migration assay

Cells were seeded (1 × 10^5^ cells/mL) in 300 μL per cell culture insert (cell culture insert for 24-well plates, 8.0 μm pore size, Falcon™ ThermoFisher Scientific, Wilmington, DE) and 800 μL growth medium with 10% FCS were added to the lower chamber. The assay was further processed as described [35].

### Invasion assay

The cell culture inserts were coated with Matrigel (1 mg/mL) and cells were seeded and processed as described in the transwell migration assay.

### Spheroid growth assay

5 × 10^3^ cells were seeded in duplicates in serum-free DMEM/Ham’s F-12 medium (Biochrom, Germany) supplemented with 20 ng/mL basic fibroblast growth factor (bFGF Eubio, Austria), 20 ng/mL epidermal growth factor (EGF; Sigma) and 2% B27 supplement (PAA Laboratories, Austria) in ultra-low attachment 24-well plates (Corning, NY) and further processed as described [36]. Re-differentiation was tested by re-plating sphere-derived single cells in 24-well plates with IMDM medium. After 7 days viable, adherent cell clones were fixed and stained with crystal violet for densitometric quantification.

### Protein isolation and western blotting

Total protein fractions were extracted and processed for Western blotting as described [37, 38] using the following primary antibodies: CD44 (156-3C11) monoclonal mouse antibody (Cell Signaling Technology, Beverly, MA), Integrin αν (Cell Signaling), Integrin α5 (Cell Signaling) and ß-actin monoclonal mouse AC-15 (Sigma, Vienna, Austria). Mouse peroxidase-labelled secondary antibodies (Santa Cruz Biotechnology) were used at working dilutions of 1:10 000.

### RNA isolation

Total RNA was isolated with Trizol reagent according to standard protocols. RNA quantity and quality were determined by Nanodrop measurements (Nanodrop 1000, Thermo Fisher Scientific). Quantity ranged between 150 and 400 ng/μL. All samples had a 260/280 ratio of > 1.8.

### Real-time PCR

500 ng of RNA were reverse transcribed into cDNA. For real-time PCR 10 ng were used for each amplification reaction (performed in triplicates). Real-time PCR was performed as described [39]. Expression levels of *CD44* all, *CD44* standard and *CD44* v6 mRNA levels were determined using the Maxima SYBR Green/ROX qPCR Mastermix (Thermo Fisher Scientific) with *ACTB* serving as a reference gene. *ACTB* primer sequence: *ACTB* sense: 5’-GGATGCAGAAGGAGATCACTG- 3’ and *ACTB* antisense: 5’-CGATCCACACGGAGTACTTG- 3’. *CD44* all sense: 5'- CGGACACCATGGACAAGTTT and *CD44* all antisense: GAAAGCCTTGCAGAGGTCAG- 3' [40]. *CD44* standard sense: 5'- AGCAGCGGCTCCTCCAGTGA and *CD44* standard antisense: CCCACTGGGGTGGAATGTGTCT -3' [40]. *CD44*v6 sense: 5'- CTGAAGACATCTACCCCAGCAAC and *CD44*v6 antisense: TTGCCAAACCACTGTTCCTTC -3' [41]. Integrin α5 primer sequence: sense: 5'- TGCAGTGTGAGGCTGTGTACA- 3' and antisense: GTGGCCACCTGACGCTCT- 3'. Integrin αv primer sequence: sense: 5'- AATCTTCCAATTGAGGATATCAC -3' and antisense:

AAAACAGCCAGTAGCAACAAT-3'. *CD44* all, *CD44* standard, *CD44*v6, integrin α5 and integrin αv mRNA expression was quantified by the comparative Ct method using *ACTB* as a reference gene. Experiments were performed twice delivering comparable results.

### Subcutaneous and intravenous OS xenografts

Animal experiments were performed based on an authorization by the Ethics committee of the Medical University of Vienna and Austrian Ministry for Science and Technology and followed the guidelines of the Federation of Laboratory Animal Science Associations (FELASA) as well as the Arrive guidelines for animal care and protection. Planning of the experiments considered in all cases the strategies to replace, reduce, and refine (“3R”). Endpoints were excessive tumor burden (>1.5cm diameter), ulceration or animal weight loss (>15% of pre-treatment weight), in accordance with the guidelines for the welfare and use of animals in cancer research [42]. Subcutaneous tumor growth was initiated by injection 10^6^ cells of the primary OS cell line U-2 OS or the sublines U-2 OS/M1, U-2 OS/M2, Saos-2, and Saos-LM7 into the right flank of 6-8 weeks old SCID/BALBc male mice (Harlan Winkelman). Each experimental group contained 4 mice. Body weight and tumor size were determined three times per week using a Vernier calliper [39]. Intravenous injection of the OS cell lines U-2 OS, U-2 OS/M1, U-2 OS/M2, Saos-2 or Saos-LM7 (10^5^ cells each) into the tail vein was used to evaluate the lung colonizing activity of the different cell models.

### Histology and immunohistochemistry

Tumor, lung, liver, and kidney were removed, fixed in buffered formalin and embedded in paraffin and further processed as described [38]. Slides were incubated for 60 min at room temperature with CD44 (CD44 Std./HCAM Ab-4 antibody, 1:100, Thermo Fisher Scientific) and Ki-67 antibodies (1:100; Dako, Agilent Technologies). The percentage of CD44-positively stained areas in comparison to the total tumor tissue areas, and Ki-67-positive cells in comparison to the total cell number were evaluated by Definiens analyses (Definiens AG, Munich, Germany).

### Array comparative genomic hybridization (aCGH)

aCGH analyses were performed as published [33] using 4x44K oligonucleotide-based microarrays (Agilent Technologies). Labelling and hybridization procedures were carried out according to protocols provided by the company. For direct comparison of U-2 OS, U-2 OS/M1, and U-2 OS/M2, indirect aCGH was performed: U-2 OS (instead of normal human reference DNA) was labelled with Cy3 and U-2 OS/M1 or U-2 OS/ M2 cells with Cy5.

### Whole genome gene expression analysis

Whole genome gene expression array analyses were performed as described in [33, 43] using 4x44K whole genome oligonucleotide-based gene expression arrays (Agilent Technologies).

### Silencing of CD44

For transient gene silencing cells were seeded (3 × 10^5^ per well) in six well plates and incubated for 24 hours before transient transfection using Lipofectamin 2000 (Invitrogen, Carlsbad, CA) with a final concentration of 2 μg shRNA for *CD44* (shCD44-2 pRRL; Addgene, Cambridge, MA) or 2 μg empty vector control (shLuc pRRL; Addgene) as described [44]. The extent of *CD44* knockdown was assessed by quantitative real-time PCR and Western blot analyses 72 h post transfection, as described above.

### Gene set enrichment analysis (GSEA)

GSEA was performed as previously described [45] to assess gene ontology terms with altered expression in parental as compare to hyper-metastatic cell models (U-2 OS/M1vs U-2 OS and U-2 OS/M2 vs U-2 OS).

### Statistical analysis

Statistical analysis was performed using GraphPad Prism 5.0 software (GraphPad Software Inc., La Jolla, CA). All experiments were carried out independently at least three times. All data are expressed as mean ± S.D. Statistical significance of differences was analysed by using Students t-test or one-way ANOVA as appropriate followed by Bonferroni post-tests. A *p*-value < 0.05 was considered statistically significant. Throughout the study the following classification is used: ^*^, *p* < 0.05; ^**^, *p* < 0.01 ^***^, *p* < 0.001.

## SUPPLEMENTARY MATERIALS FIGURES


