# Supplementary material for: CD44 drives aggressiveness and chemoresistance of a metastatic human osteosarcoma xenograft model
Source: Oncotarget. 2017 Dec 9;8(69):114095–108. doi: 10.18632/oncotarget.23125 (PMC5768389; doi:10.18632/oncotarget.23125)
Supplement: Supplementary file 1 [file oncotarget-08-114095-s001.pdf]

CD44 drives aggressiveness and chemoresistance of a metastatic human osteosarcoma xenograft model

SUPPLEMENTARY MATERIALS

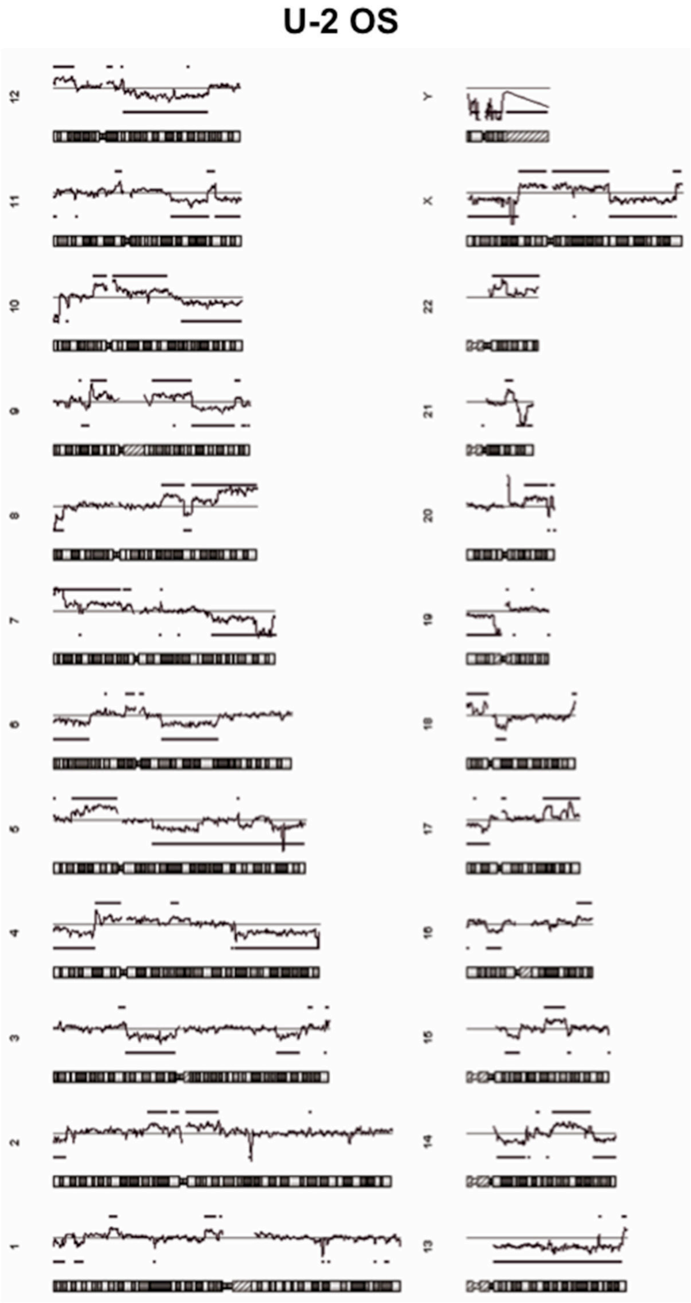

Supplementary Figure 1: DNA gains and losses in the parental U-2 OS cell line analysed by aCGH. Direct aCGH was performed of U-2 OS-derived DNA (Cy5 labeled) as compared to normal human reference DNA (Cy3 labeled). Gene dose changes of the primary osteosarcoma cell model U-2 OS are shown (bars to the right depict gains, bars to the left losses).

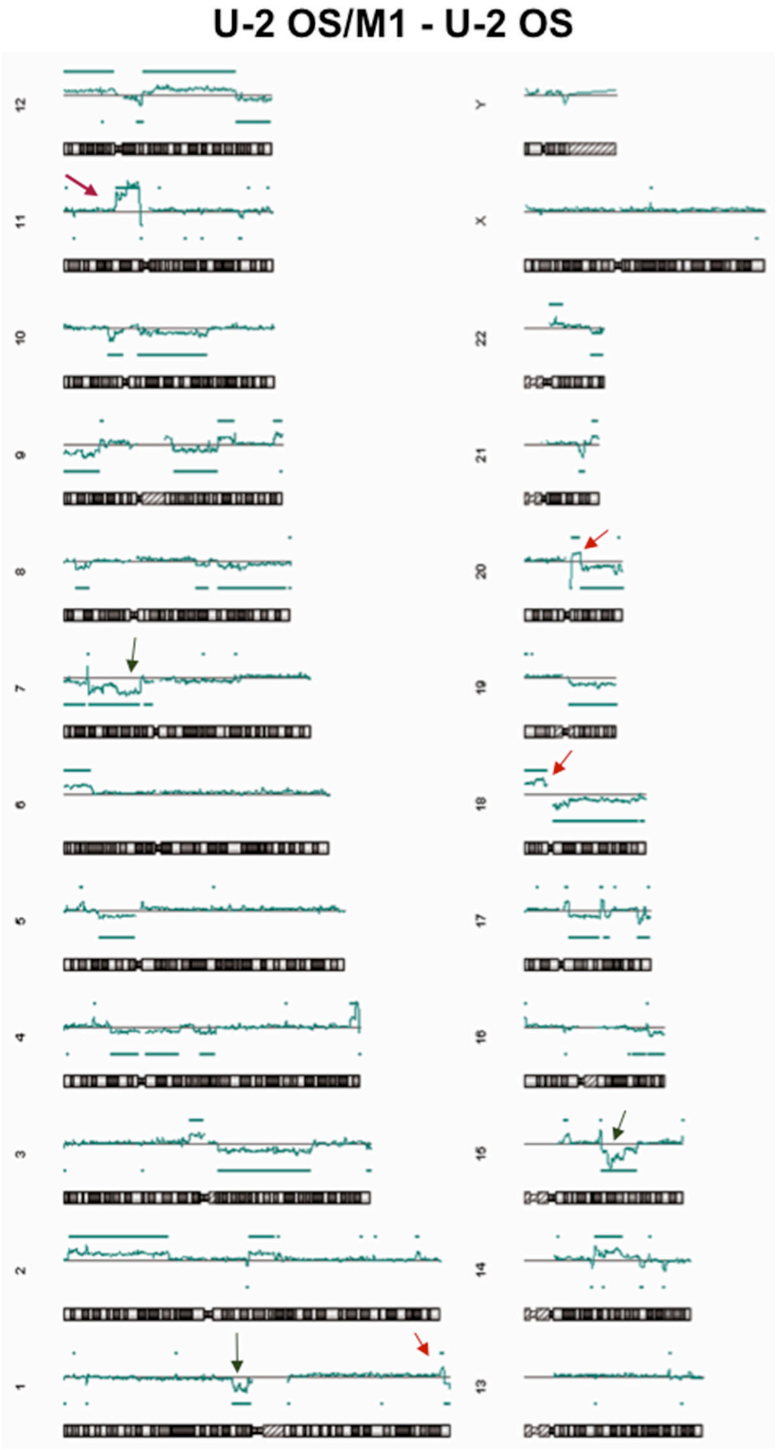

Supplementary Figure 2: DNA gains and losses in the hyper-metastatic OS cell line U-2 OS/M1 as compared to the parental U-2 OS cells. Indirect aCGH was performed by hybridizing U-2 OS/M1-derived versus U-2 OS-derived DNA. Interesting gains and losses described in the text are marked by dark red and dark green arrows, respectively. The gene locus for *CD44* is marked by a bold dark red arrow.

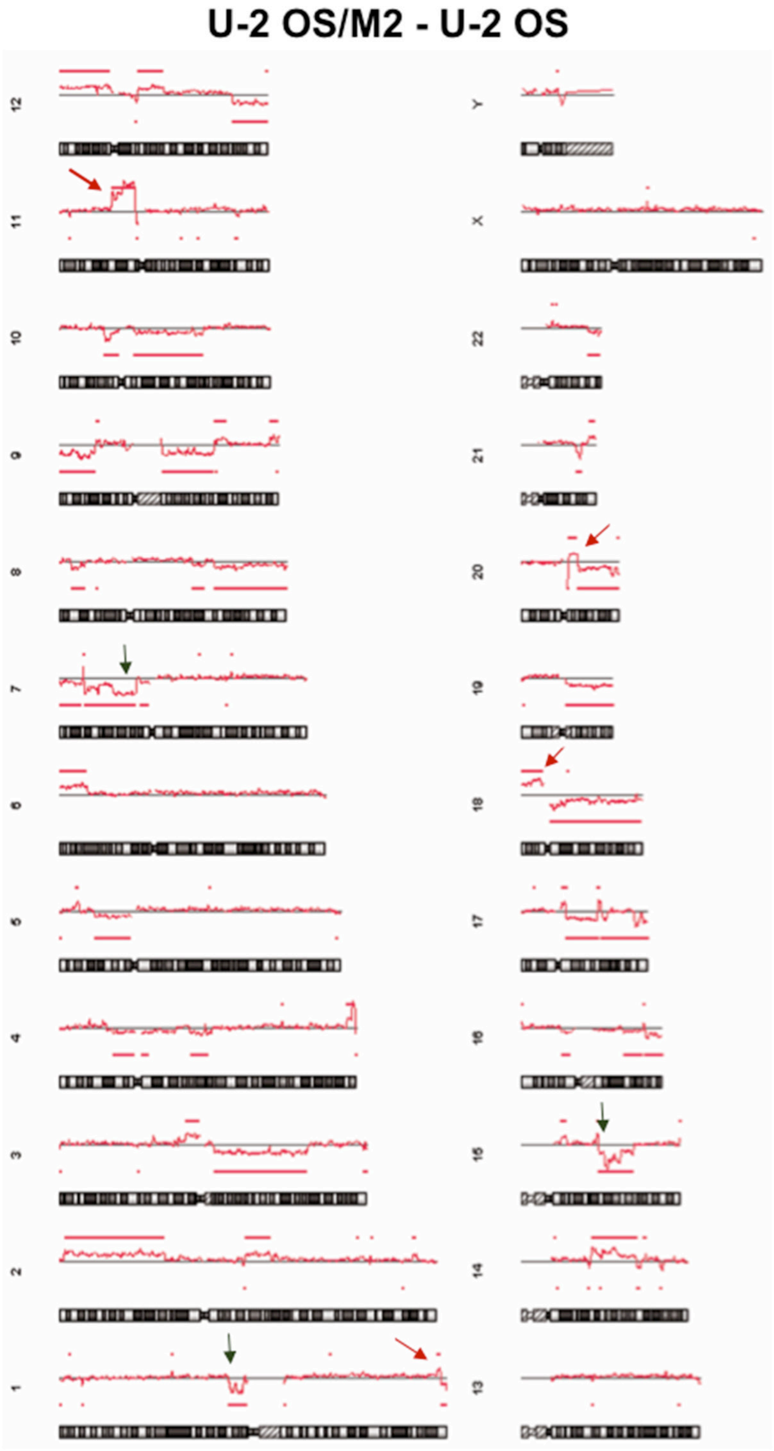

Supplementary Figure 3: DNA gains and losses in the hyper-metastatic OS cell model U-2 OS/M2 as compared to the parental U-2 OS. Indirect aCGH was performed by hybridizing U-2 OS/M2-derived versus U-2 OS-derived DNA. Interesting gains/ losses described in the text are marked by dark red and dark green arrows, respectively. The gene locus for *CD44* is marked by a bold dark red arrow.

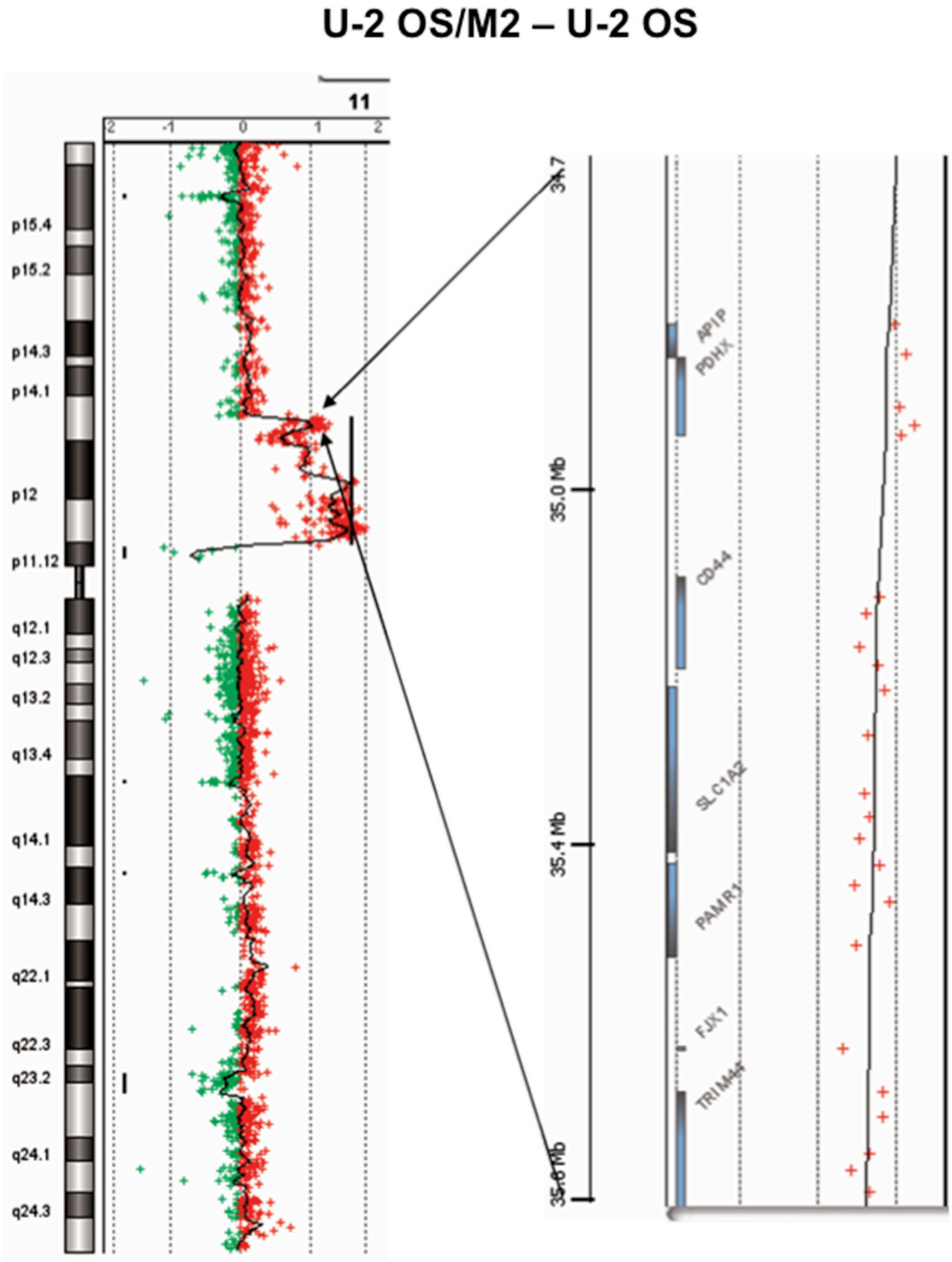

Supplementary Figure 4: Altered gene dose level of *CD44* in the hyper-metastatic OS cell model U-2 OS/M2 as compared to U-2 OS. Gains and losses of the entire chromosome 11 of U-2 OS/M2 as compared to U-2 OS cells are shown (left panel) and a detailed view of the *CD44* gene locus on 11p13 is depicted (right panel).

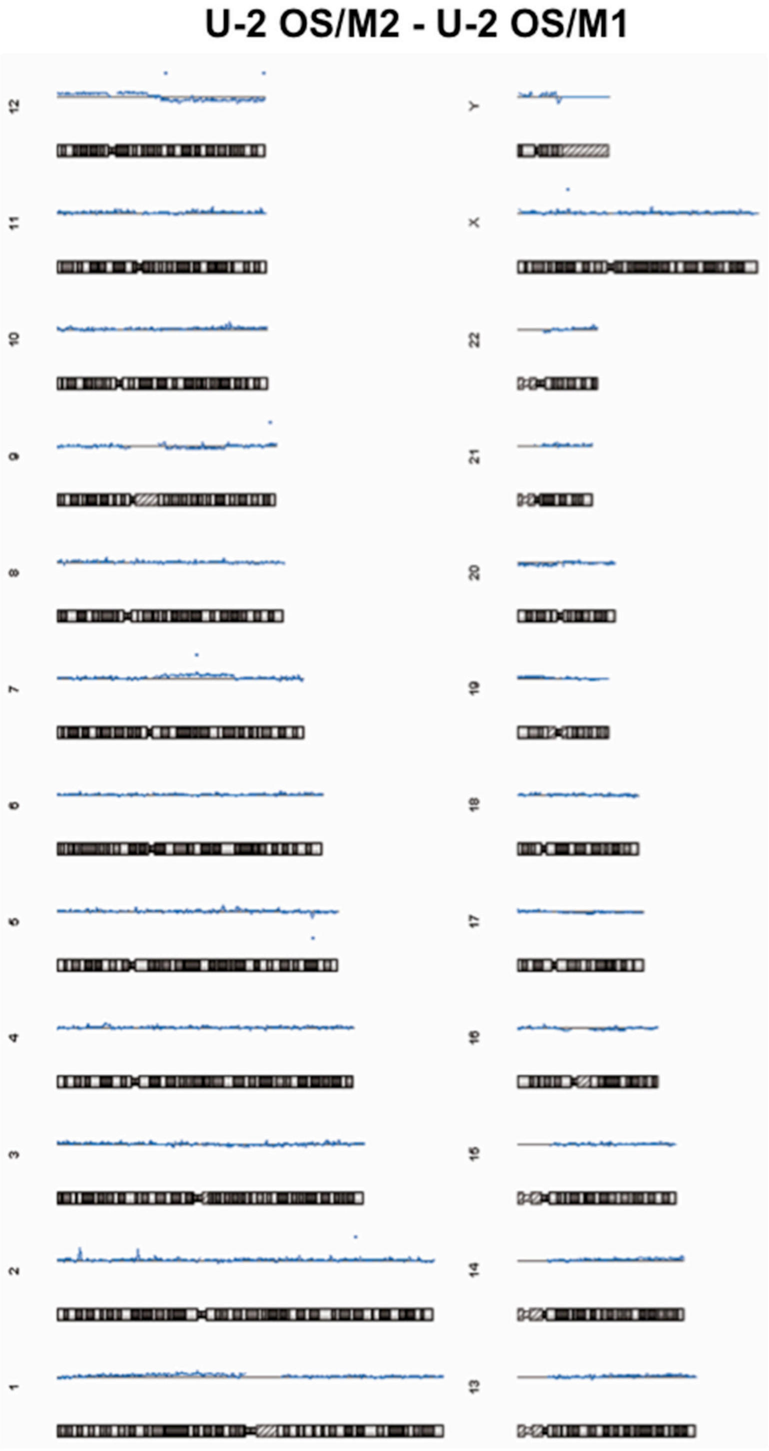

Supplementary Figure 5: Indirect aCGH comparing DNA gains and losses of the hyper-metastatic U-2 OS/M2 to U-2 OS/M1 subclones. Indirect aCGH was performed by hybridizing U-2 OS/M1-derived versus U-2 OS/M2-derived DNA.

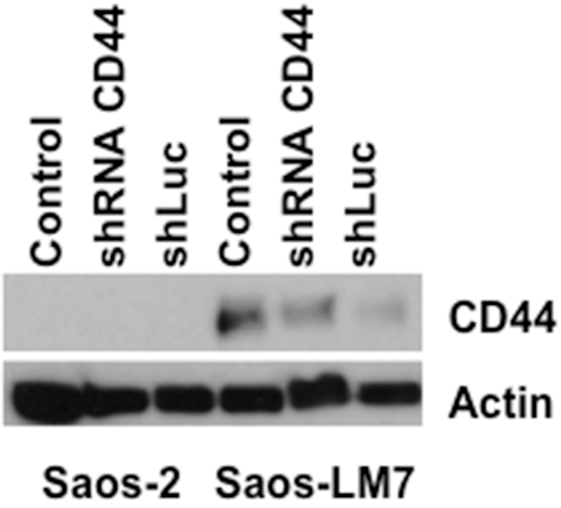

Supplementary Figure 6: *CD44* mRNA and protein expression levels in OS cells. *CD44* protein expression levels were determined by Western blot analysis in the indicated cell lines either untransfected (control) or transfected with shRNA targeting *CD44* mRNA (shRNA CD44) or shLuc vector control (shLuc).

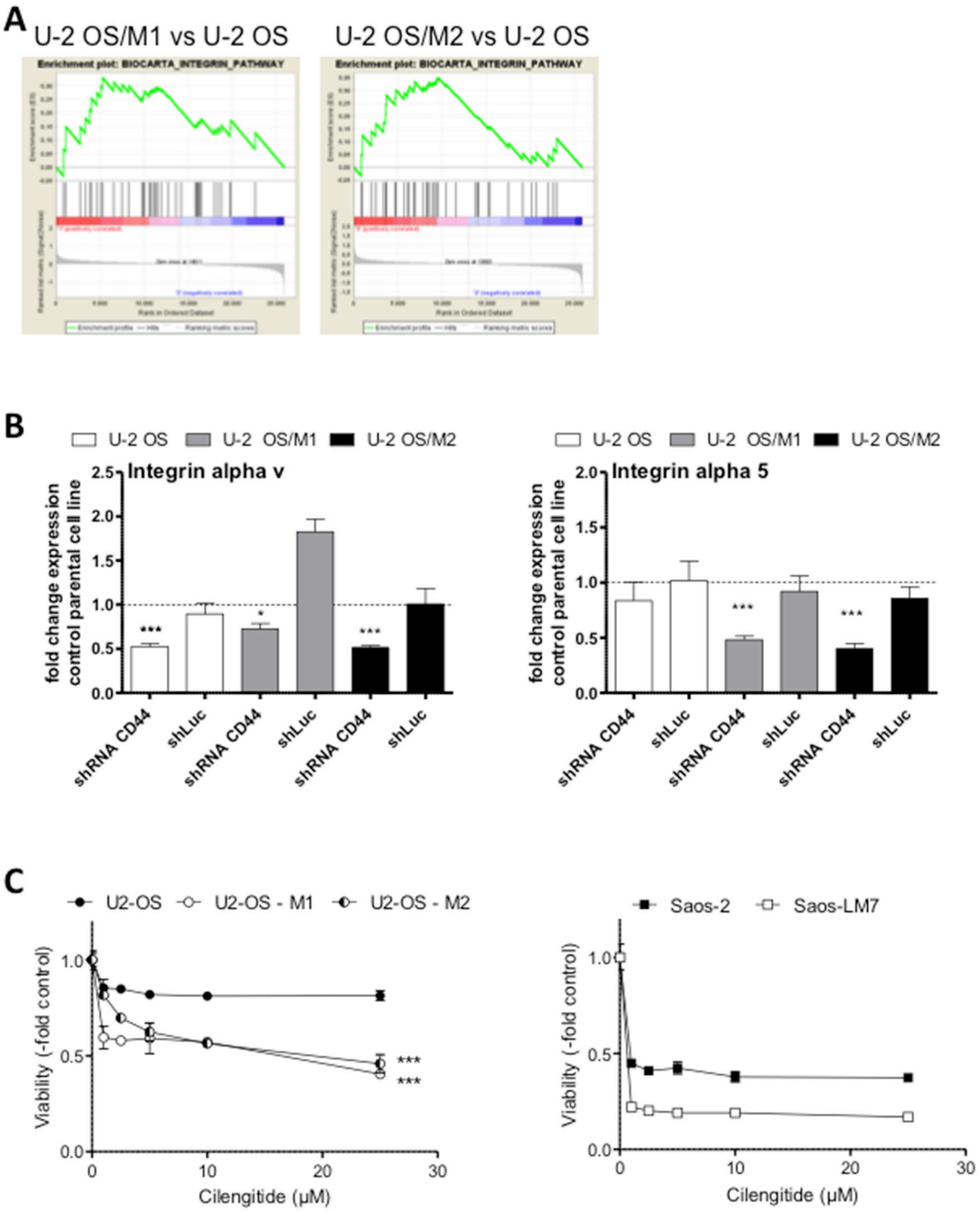

Supplementary Figure 7: Impact of Integrins on OS metastases. (A) GSEA indicated an enrichment of the Biocarta term "integrin pathway" in the hyper-metastatic cell lines U-2 OS/M1 and U-2 OS/M2 when compared to the parental cell line **(B)** Integrin αv and Integrin α5 mRNA expression was determined by real-time PCR with the indicated primer sets. Two experiments were performed in triplicates. **(C)** The effect of integrin blockade by cilengitide at the indicated concentrations was tested by MTT-based survival assays at 72 h exposure time. Two experiments were performed in triplicates. One-way ANOVA with Bonferroni's post hoc test; * $p < 0.05$; *** $p < 0.001$.

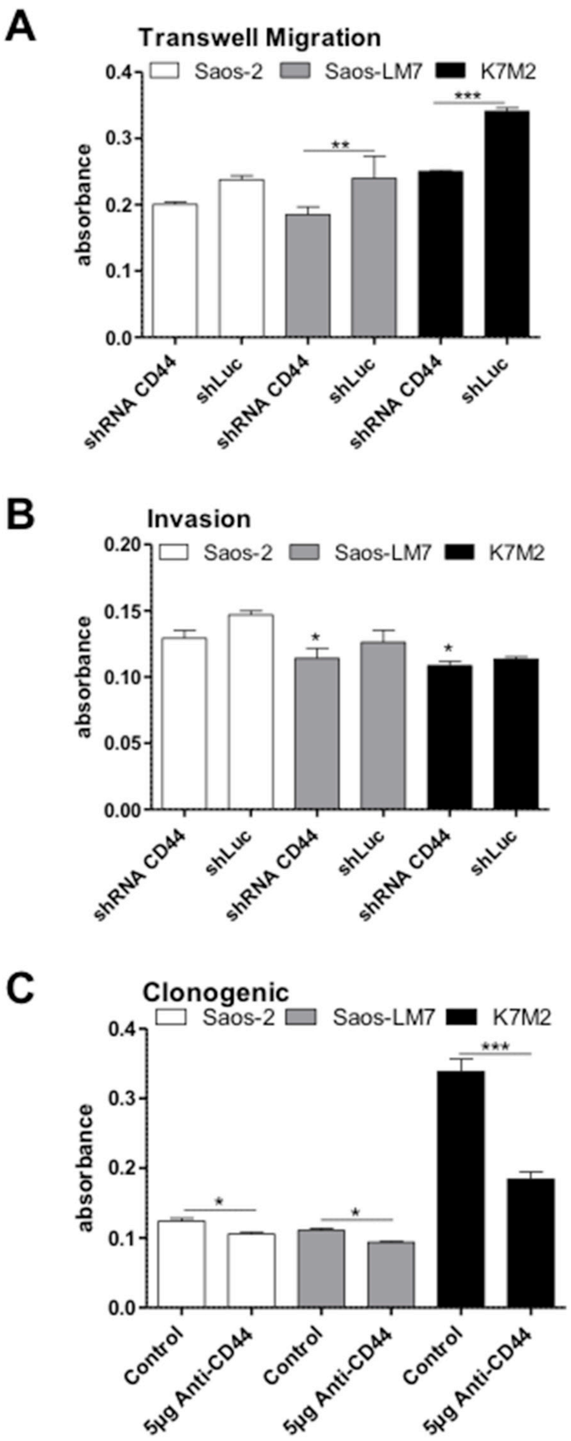

Supplementary Figure 8: Impact of *CD44* gene knockdown on OS cell migration, invasion and clonogenicity. (A) Transwell migration assays were performed for 48 hours under standard culture conditions. Cell migration to the lower side of the membrane was determined by densitometric quantification of crystal violet staining. Three experiments were performed in duplicates. **(B)** Invasion assays were performed with matrigel covered filters for 48 hours and evaluated as under A. Three experiments in duplicates were performed. Densitometric quantification of the crystal violet stained cells are shown. **(C)** Clonogenic assays were performed under standard culture conditions with and without 5 µg anti-CD44 antibody for 7 days. Clonogenicity was determined by densitometric quantification of crystal violet staining. Two experiments were performed in duplicates. One-way ANOVA with Bonferroni's post hoc test; * $p < 0.05$; ** $p < 0.01$; *** $p < 0.001$.

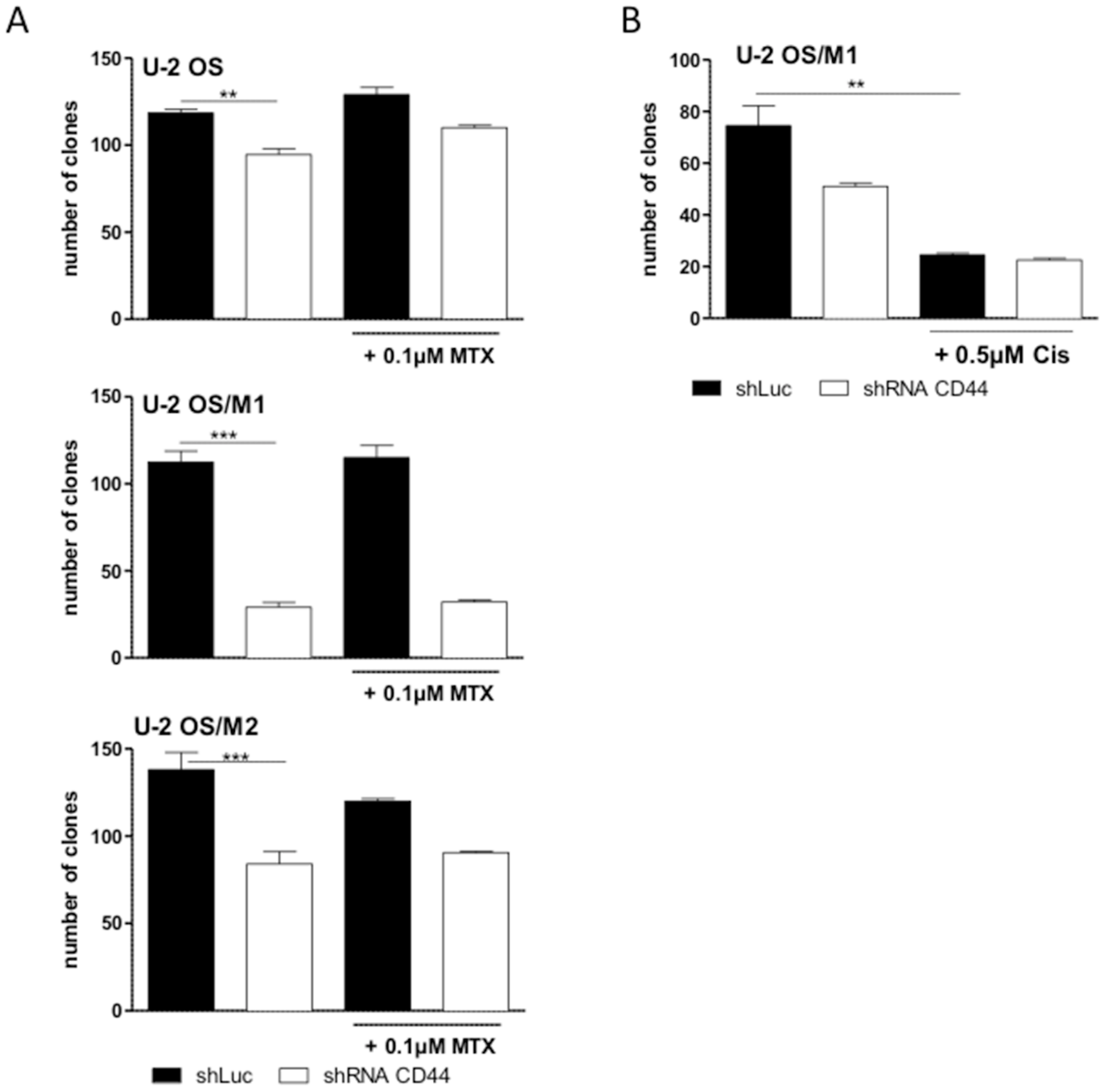

Supplementary Figure 9: Impact of *CD44* gene knockdown on chemotherapy resistance. **(A)** Clonogenic assays were performed under standard culture conditions with and without 0.1 µM methotrexate for 7 days. **(B)** Clonogenic assays were performed under standard culture conditions with and without 0.5 µM cisplatin for 7 days. Means of three experiments in duplicates are depicted. Densitometric quantification of the crystal violet stained cells are shown. One-way ANOVA with Bonferroni's post hoc test; ** $p < 0.01$; *** $p < 0.001$.

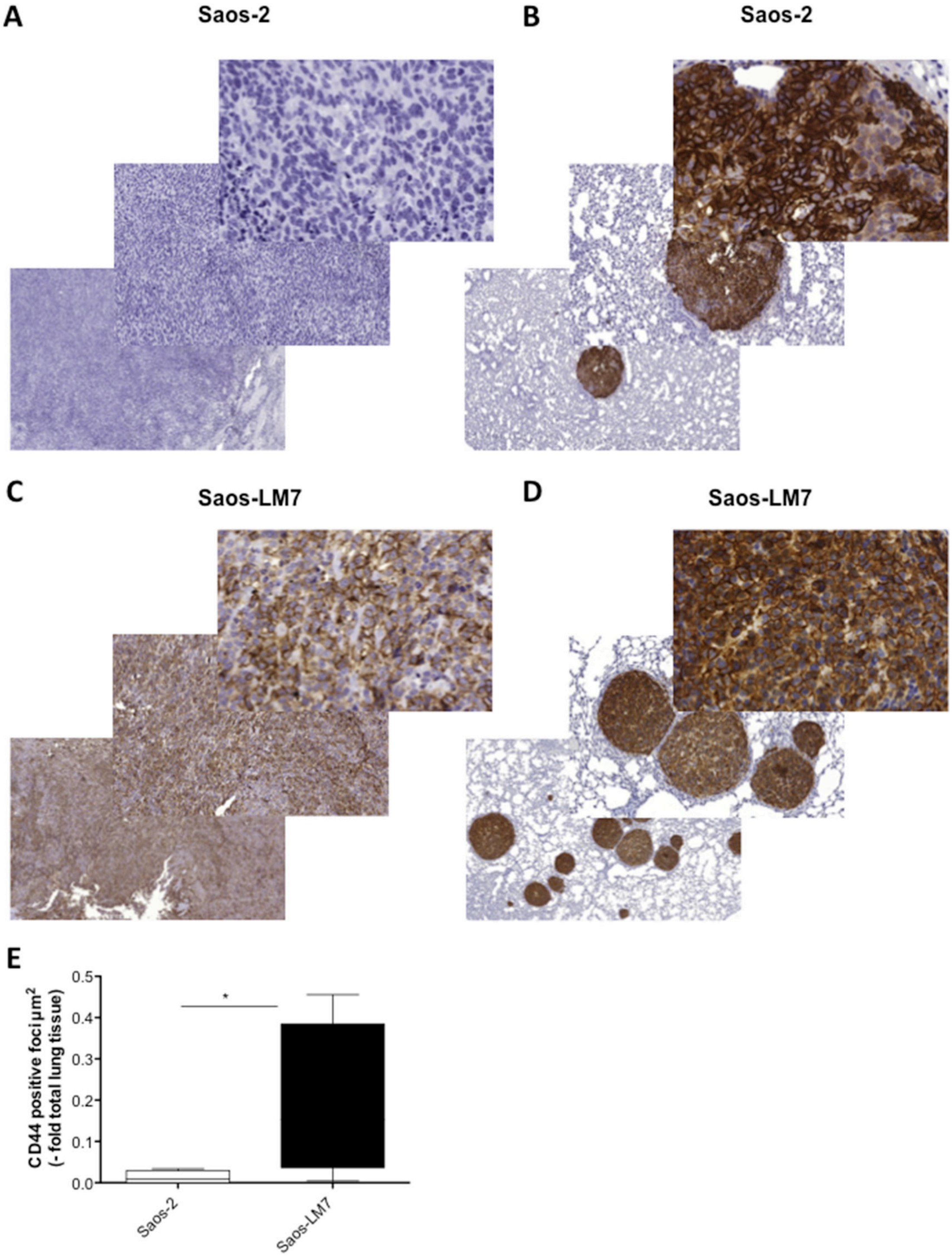

Supplementary Figure 10: CD44 expression in lung metastases and subcutaneous tumors of xenografted OS cells. Tissue sections of subcutaneous OS xenografts from Saos-2 and Saos-LM7 (**A, C**, respectively) and tail vein injection-induced lung metastases from Saos-2 and Saos-LM7 (**B, D**, respectively) were analysed immunohistochemically. Sections were stained for CD44. **(E)** Quantification of CD44 positive metastatic foci in the lung obtained after intravenous injection normalized to the total lung tissue (in μm^2). Students t-test; $^* p < 0.05$.
